# Cladribine Tablets for the First-Line Treatment of Relapsing-Remitting Multiple Sclerosis: An Evidence Review Group Perspective of a NICE Single Technology Appraisal

**DOI:** 10.1007/s40273-018-0718-2

**Published:** 2018-10-16

**Authors:** Tosin Lambe, Rui Duarte, James Mahon, Sarah Nevitt, Janette Greenhalgh, Angela Boland, Sophie Beale, Eleanor Kotas, Joanne McEntee, Ian Pomeroy

**Affiliations:** 1 Liverpool Reviews and Implementation Group, University of Liverpool, Whelan Building, Liverpool, L69 3GB UK; 2 Coldingham Analytical Services, Berwickshire, UK; 3 North West Medicines Information Centre, Liverpool, UK; 4 The Walton Centre NHS Foundation Trust, Liverpool, UK

## Abstract

As part of the single technology appraisal process, the National Institute for Health and Care Excellence invited Merck to submit evidence for the clinical and cost effectiveness of cladribine tablets (cladribine) for the treatment of relapsing-remitting multiple sclerosis (RRMS). Rapidly evolving severe (RES) and sub-optimally treated (SOT) RRMS were specified by the National Institute for Health and Care Excellence as subgroups of interest. The Liverpool Reviews and Implementation Group at the University of Liverpool was the Evidence Review Group. This article summarises the Evidence Review Group’s review of the company’s evidence submission for cladribine and the Appraisal Committee’s final decision. The final scope issued by the National Institute for Health and Care Excellence listed the following disease-modifying treatments as comparators: alemtuzumab, daclizumab, fingolimod and natalizumab. At the time of the company submission, a licence was anticipated for low-dose cladribine. The main clinical evidence (the CLARITY trial) in the company submission focused on the efficacy of low-dose cladribine vs. placebo. The CLARITY trial showed a statistically significant reduction in relapse rate for cladribine in the RES-RRMS subgroup (*n* = 50) but not in the SOT-RRMS subgroup (*n* = 19). Cladribine showed a numerical, but not a statistically significant, advantage in delaying disability progression at 6 months in the RES-RRMS subgroup. Disability progression benefits could not be estimated for those in the SOT-RRMS subgroup because of few events. The Evidence Review Group’s main concern regarding the clinical evidence was the small sample size of the subgroups. To compare the effectiveness of cladribine to other disease-modifying treatments, the company conducted network meta-analyses, which showed cladribine and its comparators to be equally effective. The Evidence Review Group considered the results of the disease-modifying treatments to be unreliable because few trials were in the network. The company’s cost-effectiveness evidence showed cladribine to be cheaper and more effective than other disease-modifying treatments in the RES-RRMS arm and the SOT-RRMS arm. The results were most sensitive to treatment effect on disability progression at 6 months. The Evidence Review Group was concerned that there was insufficient evidence to conclude that cladribine was superior to placebo in delaying disability progression. The Evidence Review Group amended the company’s economic model to allow alternative estimates for the treatment effect of cladribine and its comparators on relapse rate and disability progression at 6 months. The Evidence Review Group made other changes to the company model. After implementing all the amendments, cladribine remained cost effective in the RES-RRMS and SOT-RRMS subgroups. The Appraisal Committee recognised the uncertainty in the available data but concluded that cladribine could be considered a cost-effective use of National Health Service resources.

## Key Points

**Table:** 

The European Medicines Agency withdrew the marketing authorisation for daclizumab over safety concerns in 2018 and the National Institute for Health and Care Excellence (NICE) has subsequently withdrawn its guidance on daclizumab for treating relapsing-remitting multiple sclerosis (RRMS).
Before NICE withdrew its guidance on daclizumab, Merck submitted clinical and cost-effectiveness evidence to NICE to support the use of cladribine in patients with rapidly evolving severe RRMS and sub-optimally treated RRMS in the UK. The comparators were alemtuzumab, daclizumab, fingolimod and natalizumab.
An independent Evidence Review Group critiqued the submission and concluded that the available evidence has not sufficiently demonstrated cladribine to be clinically more effective or cost effective compared to its comparators.
The NICE Appraisal Committee considered that while there was considerable uncertainty around the available evidence, cladribine was likely to represent a cost-effective use of National Health Service resources.

## Introduction

The National Institute for Health and Care Excellence (NICE) is an independent organisation whose remit is to provide national guidance to the National Health Service (NHS) in England and Wales on a range of clinical and public health issues, including the appraisal of new health technologies. The NICE single technology appraisal (STA) process is designed for the appraisal of a single health technology for a single indication shortly before or after a UK marketing authorisation is granted for the technology. NICE develops a document (the final scope) that sets out the questions potential appraisals should address. The scope specifies the population and subgroups of interest, potential comparators to technology, health outcome measures and other specific considerations.

As part of the STA process, the company submits evidence on the clinical and cost effectiveness of the technology, including a de novo economic model. The evidence necessary to address the scope of the appraisal is expected to originate from a single company or any of its associated companies [1]. An external independent organisation, known as the Evidence Review Group (ERG) provides a critique of the company’s submission (the ERG report). The NICE Appraisal Committee (AC) considers the company’s submission (CS), the ERG report, and testimonies from clinical experts and stakeholders to determine whether the technology represents clinical and cost-effective use of NHS resources. All stakeholders and the public have an opportunity to comment on the preliminary guidance issued by NICE in the form of an appraisal consultation document, after which the AC meets again to produce the final guidance (final appraisal determination). The final guidance constitutes a legal obligation for NHS providers in England and Wales to provide a technology that is approved within its licensed indication [2].

This article presents a summary of the ERG report by the Liverpool Reviews and Implementation Group at the University of Liverpool for the STA of cladribine tablets (cladribine) for the management of patients with multiple sclerosis (MS). Merck was the sponsoring company for this STA. Full details of all documents relevant to this appraisal (including the appraisal scope, ERG report, company and consultee submissions, NICE guidance and comments on each of these) can be found on the NICE website [3].

## Background and Decision Problem

### Background

Multiple sclerosis is the most common debilitating neurological disease in young adults [4]. The disease is characterised by autoimmune-mediated inflammation, demyelination and the development of plaque lesions in the central nervous system resulting in progressive disability [5]. About 85% of patients diagnosed with MS initially present with a relapsing-remitting (RRMS) disease pattern. For these patients, episodes of acute disease exacerbation (relapse) interrupt periods of partial or complete recovery (remission) [5].

The Expanded Disability Status Scale (EDSS) is used to quantify the disability of patients living with MS. The time it takes to reach an EDSS score of 6, defined as a disability level at which patients require assistance to walk, ranges from 15 to 32 years from onset of the disease. Many factors, such as age at onset of the disease and relapse rate, affect disease progression for patients with RRMS [6]. The disease course for patients with RRMS is more aggressive in those who are categorised as having high disease activity RRMS (HDA-RRMS) [7]. This group of patients includes those with rapidly evolving severe (RES) RRMS despite adequate treatment and others with RRMS who are sub-optimally treated (SOT) [8]. Some patients with RRMS can progress to develop secondary progressive multiple sclerosis (SPMS) within 20 years following disease onset, where progressive neurodegeneration and permanent disability persist irrespective of exacerbation episodes. Highly active relapsing MS is another term used in clinical practice to describe a broad population that comprises patients with HDA-RRMS and the relapsing form of SPMS. A noteworthy point is that patients with MS are grouped into these populations based on a combination of their clinical symptoms and the number of plaque lesions in the brain following detection with magnetic resonance imaging (MRI), and the number of brain lesions only has a moderate correlation with clinical symptoms [9].

Currently, there is no cure for MS. Consequently, the main goals of clinicians managing patients with RRMS are to reduce the frequency and severity of relapses and to delay disability progression by prescribing disease-modifying therapies (DMTs). NICE recommended natalizumab [10] in 2007 and fingolimod [11] in 2012 as therapies for patients with RES-RRMS and SOT-RRMS, respectively. In 2014, [12] NICE recommended alemtuzumab for treating patients with active RRMS, which includes patients with RES-RRMS and SOT-RRMS. In 2017, daclizumab was recommended for use in patients with RES-RRMS and patients with previously treated for active RRMS (that is, including patients with SOT-RRMS) in whom alemtuzumab is either unsuitable or contraindicated [13]. The European Medicines Agency (EMA) withdrew marketing authorisation for daclizumab in 2018 following reports of serious inflammatory brain disorders, including three fatalities worldwide [14]. The EMA was also concerned that treatment with daclizumab may be linked to immune reactions in other organs, and that the drug should not be commenced in new patients [14]. NICE has since withdrawn its guidance on daclizumab for treating RRMS.

### Decision Problem

The intervention considered in this appraisal was cladribine. The mechanism by which cladribine exerts its therapeutic effects in MS is not fully clear but its effect on B and T lymphocytes interrupts immune events that exacerbate MS. A unique feature of cladribine is discontinuous immunosuppression: a phenomenon whereby periods of lymphocyte depletion during treatment are followed by a gradual repopulation back to normal levels resulting in durable efficacy well beyond the period of treatment [15]. The implication is that cladribine (an oral medication) can be given in two cycles that are 12 months apart with no treatment in between [15]. The company stated that the infrequent dosing and the oral administration route of cladribine coupled with the added benefit of not requiring monitoring above the standard care represents an innovation [16].

Cladribine is licensed in Europe for use in adults with highly active relapsing MS. Previously, the company received two negative opinions in response to its marketing authorisation applications to the EMA for the treatment of patients with RRMS in 2009 and for the treatment of patients with HDA-RRMS in 2010. On these occasions, the EMA was concerned about the safety profile of treatment with cladribine. The appraisal discussed in this paper was prompted by the company’s latest application for marketing authorisation in June 2016 for which the company provided new efficacy and safety evidence. The company expected an authorisation only for adults with HDA-RRMS; however, the EMA issued a positive opinion for adults with highly active relapsing MS [17] on 22 June, 2017, 4 days before the company made this submission to NICE.

The population described in the final scope issued by NICE is adults with RRMS. The scope also sets out different comparators for (1) patients who have not had previous treatment, (2) patients who have received previous treatment, (3) patients with RES-RRMS and (4) patients with highly active RRMS despite previous treatment, which the company termed as SOT-RRMS. The company provided clinical effectiveness evidence for patients with RRMS, HDA-RRMS, RES-RRMS and SOT-RRMS. Only the RES-RRMS and SOT-RRMS subgroups were considered in the company’s economic analyses (Table 1). No evidence was provided in the CS for other subgroups specified in the final scope, especially for patients with RRMS who are planning pregnancy.

**Table 1:** Summary of populations and comparators in the final scope issued by the National Institute for Health and Care Excellence and in the company submission for the economic analysis. Source: Company submission, Table 62

Population in the final scope	Comparators in the final scope	Population considered in the economic analysis	Comparators in the economic analysis
Patients with RES-RRMS	Alemtuzumab Daclizumab Natalizumab	Patients with RES-RRMS who are able to receive alemtuzumab (RES-RRMSa)	Natalizumab Alemtuzumab
		Patients with RES-RRMS in whom alemtuzumab is contraindicated or are otherwise unable to receive alemtuzumab (RES-RRMSb)	Natalizumab Daclizumab
Patients with highly active RRMS despite previous treatment	Alemtuzumab Daclizumab Fingolimod	Patients with SOT-RRMS who are able to receive alemtuzumab (SOT-RRMSa)	Fingolimod Alemtuzumab
		Patients with SOT-RRMS in whom alemtuzumab is contraindicated or are otherwise unable to receive alemtuzumab (SOT-RRMSb)	Fingolimod Daclizumab

*RES* rapidly evolving severe, *RRMS* relapsing-remitting multiple sclerosis, *SOT* sub-optimally treated

## Independent Evidence Review Group Report

The evidence provided by the company comprised an initial submission, a cost-effectiveness model (which is commercial in confidence) and the company’s response to the ERG’s clarification requests. The ERG report [18] is a summary and a critical review of the evidence for the clinical and cost effectiveness of the technology provided by the company. The aims of the report were to:

- assess whether the evidence submitted by the company conforms to the methodological guidelines issued by NICE;
- assess whether the company’s interpretation and analysis of the evidence are appropriate;
- indicate the presence of other sources of evidence or alternative interpretations of the evidence that could help inform the development of NICE guidance.

In addition to providing this detailed critique, the ERG modified several key company model assumptions and parameters to explore the robustness of the company’s cost-effectiveness results.

### Clinical Evidence

#### Direct Evidence

At the time of writing their CS, there was no head-to-head trial comparing the effectiveness of cladribine with treatment with any of the other relevant comparators. Therefore, the main evidence that the company provided for the effectiveness of cladribine was from a placebo-controlled study, the CLARITY trial [19, 20]. The CLARITY trial was a randomised, double-blinded, multicentre phase III trial designed to investigate the efficacy of cladribine in patients with RRMS. A total of 1326 patients were randomised 1:1:1 to receive either low-dose cladribine (3.5-mg/kg cumulative dose over 2 years; *n* = 433), high-dose cladribine (5.25-mg/kg cumulative dose over 2 years; *n* = 456) or placebo over 2 years (*n* = 437). The company focused on data from the low-dose cladribine (referred to as cladribine from here on unless otherwise specified) tablet arm, for which an EMA license was anticipated. Data from the cladribine arm and the placebo arm formed the intention-to-treat (ITT) population in the CS. In terms of subgroups, the HDA-RRMS subgroup was pre-planned in the CLARITY trial, [21] but the RES-RRMS (*n* = 50) and SOT-RRMS (*n* = 19) subgroups were defined post-hoc [22] to address the subgroups described in the NICE decision problem.

In the cladribine arm, 91.9% (398/433) of participants completed the study and 91.2% (395/433) completed treatment. Eighty-seven per cent (380/437) of participants in the placebo arm completed the study, with 86.3% completing treatment. Results showed that treatment with cladribine was statistically significantly better than placebo for all outcomes addressed, when assessed using data from the ITT population and those from the HDA-RRMS subgroup (see Table 2).

**Table 2:** CLARITY trial primary and secondary efficacy outcomes

Outcome	Cladribine	Placebo	HR (95% CI)	*P* value
ITT population	*n* = 433	*n* = 437		
Qualifying ARR (95% CI)^a^	0.14 (0.12–0.17)	0.34 (0.30–0.38)	0.42 (0.33–0.53)	< 0.001
Time to first qualifying relapse K-M estimate, % (95% CI)	80.3 (76.1–83.8)	61.1 (56.2–65.6)	0.45 (0.34–0.58)	< 0.0001
Qualifying relapse-free participants at 48 weeks, *n* (%)	353 (81.5)	300 (68.6)	–	–
Time to 3-month CDP K-M estimate, % (95% CI)	85.1 (81.3–88.2)	76.3 (71.9–80.2)	0.59 (0.43–0.81)	0.0011
Participants with 3-month CDP at 48 weeks, *n* (%)	377 (87.1)	340 (77.8)	–	–
Time to 6-month CDP K-M estimate, % (95% CI)	90.6 (87.4–93.1)	83.3 (79.3–86.6)	0.53 (0.36–0.78)	0.0014
Participants with 6-month CDP at 48 weeks, *n* (%)	386 (89.1)	348 (79.6)	–	–
Time to NEDA-3 status K-M estimate, % (95% CI)	40.1 (34.5–45.6)	12.6 (8.8–17.0)	2.21 (1.88–2.61)	0.0001
HDA-RRMS	*n* = 140	*n* = 149		
Qualifying ARR (95% CI)^a^	0.16 (0.12–0.22)	0.46 (0.38–0.55)	0.35 (0.24–0.50)	< 0.0001
Time to first qualifying relapse K-M estimate, % (95% CI)	77.1 (68.8–83.5)	53.3 (44.7–61.2)	0.40 (0.26–0.61)	< 0.0001
Qualifying relapse-free participants at 48 weeks, *n* (%)	112 (80.0)	89 (59.7)	–	–
Time to 3-month CDP K-M estimate, % (95% CI)	91.0 (84.7–94.8)	71.7 (63.4–78.5)	0.28 (0.15–0.54)	0.0001
Participants with 3-month CDP at 48 weeks, *n* (%)	126 (90.0)	109 (73.2)	–	–
Time to 6-month CDP K-M estimate, % (95% CI)	95.5 (90.2–97.9)	77.7 (69.8–83.8)	0.18 (0.08–0.44)	0.0001
Participants with 6-month CDP at 48 weeks, *n* (%)	129 (92.1)	112 (75.2)	–	–
Time to NEDA-3 status K-M estimate, % (95% CI)	43.7 (35.0–52.0)	6.9 (2.8–13.6)	2.86 (2.14–3.81)	0.0002

*ARR* annualised relapse rate, *CI* confidence interval, *CDP* confirmed disability progression, *HAD-RRMS* high disease activity relapsing-remitting multiple sclerosis, *HR* hazard ratio, *ITT* intention-to-treat, *K-M* Kaplan–Meier, *NEDA-3* no evidence of disease activity at 3 months

^a^HR estimate is rate ratio

Results for the RES-RRMS subgroup were mixed, with treatment with cladribine being statistically significantly better than placebo in terms of qualifying annualised relapse rate (ARR) and time to first relapse; however, treatment with cladribine did not statistically significantly delay the time to 3-month confirmed disability progression (CDP) or time to 6-month CDP. For all but one outcome, there was no evidence that cladribine was statistically significantly better than placebo for patients in the SOT-RRMS subgroup. The exception was the post-hoc composite efficacy outcome ‘no evidence of disease activity’ (NEDA-3), which was defined as no relapses, no 3-month confirmed EDSS progression, no new or enhancing T1 gadolinium-enhancing (Gd+) lesions, and no new or enlarging T2 lesions [22, 23]. In terms of this NEDA-3, treatment with cladribine was statistically significantly better than placebo in the ITT population and in all subgroup populations.

The adverse events (AEs) reported in the CS were derived from participants in the CLARITY trial who were randomised to receive cladribine at a dose of 3.5 mg/kg (*n* = 430) or placebo (*n* = 435) and who received at least one study treatment. No AE data were provided for the RES-RRMS and SOT-RRMS subgroups. Overall, the proportion of participants reporting treatment-emergent AEs in the cladribine arm and placebo arm was similar (80.7% vs. 73.3%). The company reported that more patients in the cladribine arm experienced serious treatment-emergent AEs than in the placebo arm (8.4% vs. 6.4%).

The company included data from the CLARITY-EXT trial [24] in the CS. The CLARITY-EXT trial was a 2-year extension of the CLARITY trial, which explored whether the treatment effect observed during the CLARITY trial persisted in the absence of additional treatment. Participants who had received cladribine in the CLARITY trial were allowed to receive cladribine in the CLARITY-EXT trial. The rank preserving structural failure time model [25] and the iterative parameter estimation algorithm were used to estimate the effect of having these patients who had initially received placebo but then went on to receive cladribine in the CLARITY-EXT trial. Results from the company’s analyses suggested that the effect of cladribine was constant over the 4 years for which data were available. However, the company noted that the effectiveness of cladribine beyond 4 years remained uncertain.

#### Indirect Evidence

The company performed network meta-analyses (NMAs), where data were available, for several efficacy, safety and health-related quality of life (HRQoL) outcomes in the populations of interest (ITT, HDA-RRMS, RES-RRMS and SOT-RRMS). Data sources used within the NMAs were obtained from the published clinical literature, and from unpublished study reports for the CLARITY and PRISMS [26] trials. Forty-four studies contributed to the network. Some of the included studies had participants with a progressive disease, and did not report results separately for the RRMS-only population.

The results of the NMAs showed that the comparative data for cladribine vs. the relevant comparators within all of the subgroups were limited (Table 3). The limitation was pronounced for the SOT-RRMS subgroup as there were no comparative data for time to 3-month CDP and time to 6-month CDP. Treatment with cladribine was statistically significantly better than placebo at reducing ARR in the RES-RRMS subgroup. Cladribine also demonstrated a numerical advantage over placebo in delaying time to 3-month CDP and 6-month CDP, but these benefits were not statistically significant. In the SOT-RRMS subgroup, treatment with cladribine only showed a numerical but not a statistically significant advantage compared with placebo.

**Table 3:** Summary of the network meta-analysis results between cladribine and comparators for intention-to-treat (ITT) population and post-hoc subgroups. Source: Company submission, Appendix D, adapted from Tables 11–13

Cladribine vs.	ARR				3-month CDP at 24 months				6-month CDP at 24 months			
	ITT	HDA	RES	SOT	ITT	HDA	RES	SOT	ITT	HDA	RES	SOT
Placebo	↑	↑	↑	↑	↑	↑	↑	–	↑	↑	↑	–
Alemtuzumab	↓	↔	↓	–	↓	–	–	–	↓	↑	↓	–
Daclizumab	↔	–	↑	–	↔	–	–	–	↔	–	–	–
Fingolimod	↔	↔	↑	↔	↑	↑	↔	–	↑	–	–	–
Natalizumab	↓	↓	↓	–	↓	*–*	↓	–	↓	–	↓	–

A random-effects model was applied to the NMA for the ITT population and a fixed-effects model was applied to the NMA for the HDA-RRMS, RES-RRMS and SOT-RRMS subgroups

*ARR* annualised relapse rate, *CDP* confirmed disability progression, *HDA* high disease activity, *NMA* network meta-analysis, *RES* rapidly evolving severe, *RRMS* relapsing-remitting multiple sclerosis, *SOT* sub-optimally treated, ↑ indicates better efficacy for cladribine, ↑ indicates statistically significantly better efficacy for cladribine, ↓ indicates lower efficacy for cladribine, ↔ indicates equivalent efficacy of cladribine and comparator, *–* indicates that NMA was not feasible, either owing to a lack of connections within the networks or a lack of studies

When comparative data were available, the NMA results showed that treatment with cladribine was not statistically significantly better than any of the relevant comparators in terms of ARR, 3-month CDP and 6-month CDP. There were outcomes for which cladribine was numerically superior and at other times, the comparators were numerically superior to cladribine.

The company urged that the NMA results (Tables 3, 4) should be interpreted with caution owing to the heterogeneity between the studies with respect to study phase, eligibility criteria, baseline EDSS, treatment history and the onset of disease symptoms. The company acknowledged that the lack of published data linking the control arm of studies evaluating alemtuzumab via a network made it challenging to compare alemtuzumab to other therapies listed in the final scope issued by NICE for the treatment of patients with RES-RRMS and SOT-RRMS. Comparative 6-month CDP efficacy results were key inputs for the economic model, but published data on this outcome were particularly limited. The company, therefore, conducted an additional meta-regression analysis to attempt to generate a more robust comparison of 6-month CDP between cladribine and its comparators using guidance from a technical support document [27] produced by the NICE decision support unit.

**Table 4:** Network meta-analysis (NMA) for qualifying annualised relapse rate for cladribine vs. relevant comparators for the intention-to-treat (ITT) population and the high disease activity relapsing-remitting multiple sclerosis (HDA-RRMS) subgroup. Source: Company submission, Appendix D, Table 14

Cladribine	ITT	HDA-RRMS
	Median RR (95% CrI)	Median RR (95% CrI)
Placebo	0.42 (0.32–0.54)	0.35 (0.24–0.51)
Alemtuzumab	1.31 (0.95–1.82)	0.99 (0.59–1.66)
Daclizumab	0.92 (0.67–1.26)	^a^
Fingolimod	0.91 (0.68–1.23)	0.95 (0.58–1.54)
Natalizumab	1.24 (0.89–1.71)	1.14 (0.70–1.84)

A random-effects model was applied to the NMA for the ITT population and a fixed-effects model was applied to the NMA for the HDA-RRMS subgroup

*CrI* credible interval, *RES* rapidly evolving severe, *RR* rate ratio

^a^The NMA was not feasible, either owing to a lack of connections within the networks or a lack of studies

As all comparisons within the meta-regression were vs. placebo, the comparator interventions were compared in terms of the numerical results and overlap of credible intervals. Meta-regression results showed that treatment with cladribine was predicted to be more efficacious than alemtuzumab, but less efficacious than natalizumab and daclizumab for the RES-RRMS subgroup. Results also showed that treatment with cladribine was more efficacious than alemtuzumab and fingolimod, but less efficacious than daclizumab for the SOT-RRMS subgroup. However, none of the meta-regression results was statistically significant. The company concluded that the meta-regression predicted all comparators to be less effective for patients in the SOT-RRMS subgroup than for those with RES-RRMS and that, owing to the significant overlap in the credible intervals across all comparisons, no therapy was statistically significantly dominant.

### Critique of the Clinical Evidence and Interpretation

#### Direct Evidence

The ERG considered that the CLARITY trial was of good quality and was well conducted. The company presented effectiveness evidence for three subgroups of participants, two of which were defined post-hoc. The ERG acknowledged that the company’s post-hoc definition of subgroups was necessary to address the NICE decision problem, but noted that small subgroups reduced the statistical power of such analyses and the post-hoc definition of subgroups added to the uncertainty around the results. With only 19 patients in the SOT-RRMS subgroup, it was always going to be unlikely that any results generated using the subgroup data would have the statistical power to detect a difference between treatments. Two post-hoc efficacy outcomes (NEDA-3, and 6-month CDP) were presented in the CS. These outcomes were chosen to demonstrate prolonged efficacy in the reduction in disability progression in patients treated with cladribine compared with placebo. Clinical advice to the ERG was that NEDA-3 and CDP scores have not been validated as predictors of long-term outcome and that the result of these post-hoc analyses should, therefore, be interpreted with caution. The ERG noted that the result of ‘time to achieve NEDA-3 status’ in the SOT-RRMS subgroup should be interpreted with caution as this was the only outcome for which a statistically significant advantage to cladribine over placebo was observed.

#### Indirect Evidence

The ERG considered that the company’s general approach to undertaking NMAs was appropriate. However, the company did not provide information about the number of participants contributing to the NMAs of the key efficacy outcomes for the post-hoc subgroups, except for 6-month CDP at 24 months in the RES-RRMS subgroup. Furthermore, the ERG was unable to extract the participant numbers, participant characteristics, and the definitions used in the trials for the RES-RRMS and SOT-RRMS subgroups from the published literature of all of the trials. The ERG acknowledged that fitting random-effect models within small networks is difficult and agreed that the use of fixed-effect models may have been appropriate for the subgroups. However, as baseline characteristics and heterogeneity measures within the post-hoc subgroups were not available, the ERG noted that it was difficult to judge whether important statistical inconsistency or heterogeneity was present within the results for the subgroups; hence, it was difficult to interpret the numerical NMA results within the subgroups.

The company also performed a meta-regression. However, in light of the company’s stated objective, the ERG was not convinced that the results of the company’s meta-regression provided more robust results than those of the NMAs. The ERG noted that the meta-regression approach outlined in the NICE Decision Support Unit document [27] is used to explore treatment-covariate interactions, such as an interaction between treatment effect and baseline risk, as a source of heterogeneity. The ERG was uncertain whether the methods in the Decision Support Unit document were valid for using baseline risk estimates to predict treatment effect for specific subgroups.

### Cost-Effectiveness Evidence

**Fig. 1:**
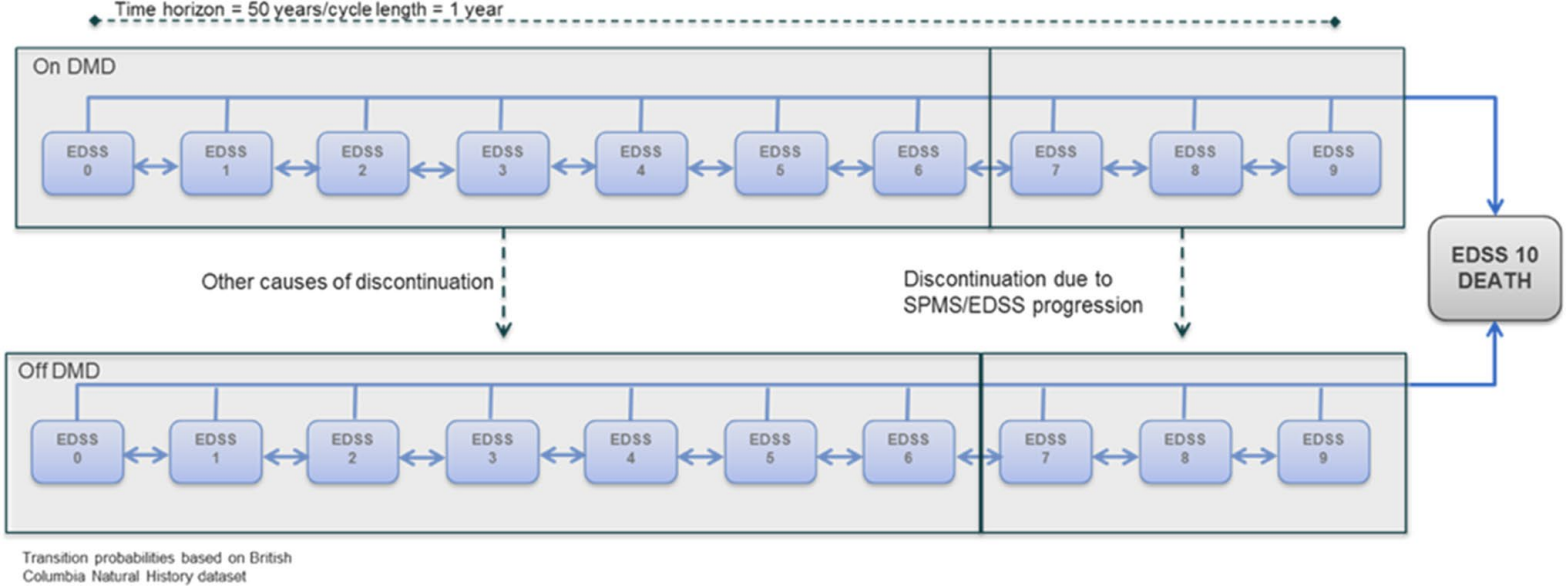
Health-state structure of the company model showing the ten Expanded Disability Status Scale (EDSS)-based health states while taking disease-modifying drugs (DMDs) or not taking DMDs. *SPMS* secondary progressive multiple sclerosis. Source: Company submission, Fig. 12

The company’s economic evaluation compared the cost effectiveness of cladribine with alemtuzumab, daclizumab, fingolimod and natalizumab (Table 1) in patients with RES-RRMS and SOT-RRMS. Using Microsoft Excel, the company built a simplified version of other economic models that, since 2005, had been submitted to NICE as part of STAs of drugs for treating MS. Previously submitted models [10–13] had a 21-health-state structure: ten EDSS-based health states for patients with RRMS, another ten EDSS-based health states for patients with SPMS and one state for all-cause mortality. The submitted company model for this appraisal, however, comprised 11 health states, having ten EDSS-based states and one additional state for death from all causes (Fig. 1). The company justified the use of the simpler model on the grounds that HRQoL is more reliably stratified by EDSS state alone, [28, 29] rather than by EDSS state and the clinical form of MS (RRMS and SPMS). The company noted that identifying and modelling the transition from the RRMS subtype into the SPMS subtype would have been challenging given that the use of SPMS-specific health states required data on SPMS-specific transition rates. The company considered the only available data on SPMS-specific transition rates (the London Ontario Registry) to be too limited.

On model entry, individuals with RES-RRMS and SOT-RRMS in the model were assigned to each of the EDSS health states based on the distribution of participants in the CLARITY trial at baseline. At the end of each 1-year cycle, there was a risk of moving to a higher EDSS state, moving to a lower EDSS state, remaining in the current EDSS state or dying. The risk of experiencing one or more acute relapses or discontinuing treatment was modelled as events within each EDSS-based health state. Individuals who discontinued DMTs were modelled to receive best supportive care.

Costs were calculated as a function of EDSS health state, number of relapses and time in each state. Included costs comprised drug cost (acquisition, administration and monitoring), hospital admission, outpatient care, relapse and informal care. Health-related quality of life for patients in EDSS 0–5 was obtained from the CLARITY trial. The EQ-5D-3L questionnaires were administered on day 1, week 24, week 48, week 72, week 96 and at each relapse evaluation during the CLARITY trial. Data from completed questionnaires were converted to utility values using the UK social tariff. The company also carried out a systematic literature review to identify relevant HRQoL data. Following an assessment of available evidence, the company used data from published studies [30, 31] for EDSS 6–9. The company reports that its approach is in line with the approach taken in previously submitted company models [10–13, 32, 33].

The model had a lifetime time horizon (50 years) and NHS and personal and social services perspectives. All costs were expressed in UK pounds sterling for the 2015/2016 price year. Health outcome was measured in quality-adjusted life-years (QALYs). The company presented results as incremental cost-effectiveness ratios (ICERs) per QALY gained, using list prices for all drugs. A 3.5% annual discount rate was applied to costs and QALYs [2]. The company carried out a wide range of deterministic sensitivity analyses for the comparison of cladribine vs. treatment with other DMTs that were within the final scope issued by NICE for RES-RRMS and SOT-RRMS.

The company’s base-case deterministic ICERs per QALY gained showed that cladribine dominated all comparators, by being more clinically effective at a lower cost. Cladribine was particularly cost effective for patients with RES-RRMS for whom alemtuzumab was either contraindicated or not tolerable. In this subgroup, cladribine delivered the largest QALY gain per patient (0.924) when compared with daclizumab, and generated the largest cost savings vs. natalizumab (£130,676). The results showed that the base-case analyses were most sensitive to variation in the effect of DMTs on 6-month CDP.

### Critique of the Cost-Effectiveness Evidence and Interpretation

The ERG considered the company’s model to be generally well structured and was satisfied with the company’s rationale for using a simplified 11 health-state model rather than a 21 health-state model. The main issues with the company model were concerns with the treatment effectiveness estimates for cladribine and its comparators, and the inclusion of costs and outcomes that were outside of the scope specified by NICE.

#### Treatment Effect Estimate for Cladribine

The company used the point estimates for ARR and 6-month CDP that were obtained from the CLARITY trial as efficacy data for cladribine. The point estimates suggested that cladribine was superior to placebo for both efficacy outcomes for the RES-RRMS and SOT-RRMS subgroups. The ERG considered that the direct evidence presented by the company supports the conclusion that cladribine is superior to placebo but only for the RES-RRMS subgroup and only for qualifying ARR. When the ERG modelled its interpretation of the CLARITY trial data for the RES-RRMS subgroup, treatment with alemtuzumab dominated cladribine, cladribine was less costly and generated fewer QALYs vs. natalizumab and daclizumab but at a cost per QALY lost that favoured the use of cladribine. Because evidence from the CLARITY trial suggests that treatment with cladribine is not statistically significantly better than placebo in terms of ARR or 6-month CDP for patients with SOT-RRMS, the ERG considered that there was no robust basis on which to construct an economic model for that subgroup.

The ERG expressed concerns about the robustness of the methods that were used to derive the comparative estimates on ARR (from the NMA) and 6-month CDP (from the meta-regression). The company had modelled the effect of DMTs on progression between EDSS states by applying the hazard ratios for 6-month CDP from the meta-regression analysis to baseline transition probabilities. The ERG was concerned about the robustness of the meta-regression. However, even if the results from the company’s statistical analyses were robust for both the RES-RRMS and SOT-RRMS subgroups, the credible interval of the hazard ratio for all DMTs overlapped and the point estimates were similar.

The company modelled relapse rates as a function of time by multiplying the number of patients alive by ARR. Similar to the 6-month CDP results, the ARR results for each DMT (cladribine and it comparators) vs. placebo in the SOT-RRMS subgroup showed that the point estimates for the comparators were similar and resided within the credible intervals of every other DMT. The picture was slightly less clear for the RES-RRMS subgroup. The ARR point estimate for cladribine, alemtuzumab and daclizumab compared to placebo were further apart than those for the SOT-RRMS subgroup but still resided in each other’s credible intervals. The point estimate for natalizumab only resided in the alemtuzumab credible interval, but the credible interval for natalizumab nonetheless overlapped with those of other DMTs.

The ERG considers that, in situations where confidence/credible intervals overlap and point estimates are similar, the appropriate approach is to assume equal efficacy for all treatment options. As such, the ERG assumed that the 6-month CDP hazard ratios for all comparators were the same as those generated by the company’s meta-regression for cladribine. The ERG also assumed that the ARR result in the RES-RRMS subgroup was the same for all DMTs other than natalizumab. These changes had no effect on the company’s base-case cost-effectiveness results as cladribine dominated all of the other comparators.

#### Treatment Effect over Time

The company analysed the CLARITY-EXT data in an attempt to provide robust evidence about the extent to which the effect of cladribine on 6-month CDP wanes over a 4-year time horizon. In the absence of long-term follow-up data, in previous NICE appraisals of drugs for treating MS, the waning of effectiveness over time had been assumed to be the same for all DMTs, namely 100% for the first 2 years, 75% for the next 2 years and 50% for subsequent years (Table 5).

**Table 5:** Changes in drug efficacy over time that the company applied in the model. Source: Company submission, Tables 71 and 73

Years	Cladribine		Comparators	
	Value (%)	Rationale	Value (%)	Rationale
0–2	100	Analysis of pooled data from CLARITY and CLARITY-EXT trials	100	Based on approaches that were accepted by NICE in TA441 [13]
2–4	100		75	
4–5	75	Based on approaches that were accepted by NICE in TA441 [13]	75	
5+	50		50	

*NICE* National Institute for Health and Care Excellence

The waning effect estimates reported in the CS were based on the pooled 6-month CDP data from the CLARITY trial and the CLARITY-EXT trial for the ITT population, not for the RES-RRMS and SOT-RRMS subgroups. Thus, as part of the clarification process, the ERG requested further analyses of waning using 6-month CDP and ARR for the RES-RRMS and SOT-RRMS subgroups.

For the RES-RRMS subgroup, the sample size was smaller and the number of outcomes was fewer than those in the ITT population, meaning that the confidence intervals were even wider than the confidence interval for the ITT population analysis of waning. For both the ITT population and the RES-RRMS subgroup, the confidence intervals for the hazard ratios used to support no waning between years 2 and 4 were wide and included a reduction in effectiveness of 75%. The ERG interpreted this evidence to mean the waning for cladribine is the same as has been assumed for other DMTs in previous appraisals. In their clarification response, the company stated that, for the SOT-RRMS subgroup, the sample size and number of events were too small for the treatment-switching algorithm to generate credible results. They did, however, undertake the analysis and results showed that there was no evidence that the effectiveness of cladribine waned in the SOT-RRMS subgroup.

Clinical advice to the ERG was that there is almost complete uncertainty around the extent, and timing, of any waning of treatment effect for patients with RES-RRMS or SOT-RRMS (or for any patients with MS) who receive any of the DMTs included in the company model, for the period beyond 2 years. The ERG considered that the evidence provided by the company was not strong enough to merit the application of a waning effect for cladribine that is different to that used for the other DMTs. Setting all treatments to have the same waning effect (100% up to year 2, 75% over years 2–4 and 50% thereafter) had no effect on the company’s base-case cost-effectiveness results, i.e. cladribine dominated all the comparator treatments for the RES-RRMS and SOT-RRMS subgroups.

#### Other Issues

The company included informal care costs and carer disutility in its base-case analyses. The ERG considered this to be inappropriate as these are not NHS or personal and social services costs. The ERG investigated the impact of excluding carer disutility and using alternative costs derived from previous NICE submissions.

The company’s base-case analysis assumed only a single line of treatment. As modelling of treatment sequencing is beyond the remit of the ERG, the ERG considered it informative to explore time horizons significantly shorter than lifetime to reflect the facts that (1) patients are unlikely to be receiving a single treatment for life and (2) that the effectiveness data available for the DMTs are limited to, at the most, 4 years.

The company also considered that patients treated with cladribine and alemtuzumab could re-start with their respective medications after 4 years. Treatment re-initiation rates for cladribine were derived from the CLARITY-EXT trial, while those for alemtuzumab were derived from previous STAs [12, 13]. Clinical advice to the ERG was that there is no published evidence that re-initiation is effective. The ERG, therefore, considered that it was appropriate to remove re-exposure to cladribine and alemtuzumab from the base-case analyses. The ERG also considered alternative treatment discontinuation rates and AE rates for all DMTs.

None of the ERG’s changes, except for shortening the time horizon, stopped cladribine from dominating its comparators. However, the cumulative impact of the ERG’s various amendments to the company’s base-case model did affect the company’s base-case conclusion for patients with RES-RRMS. Alemtuzumab dominated cladribine, and cladribine no longer dominated daclizumab and natalizumab, with ICERs of £81,050 and £64,269 per QALY lost respectively (Table 6).

**Table 6:** Cost-effectiveness results for the comparison of cladribine vs. alemtuzumab, natalizumab, daclizumab and fingolimod for patients with rapidly evolving severe relapsing-remitting multiple sclerosis (RES-RRMS) and sub-optimally treated relapsing-remitting multiple sclerosis (SOT-RRMS): company base-case results and results generated following Evidence Review Group (ERG) revisions to the company base case

RES-RRMS	Alemtuzumab			Natalizumab			Daclizumab		
	Inc. cost	Inc. QALYs	ICER	Inc. cost	Inc. QALYs	ICER	Inc. cost	Inc. QALYs	ICER
Company base case	− £19,134	0.182	Dominant	− £130,676	0.512	Dominant	− £89,182	0.924	Dominant
ERG scenario (R1, R2–R6)	£38,423	− 1.541	Dominated	− £133,754	− 1.650	£81,050^a^	− £87,566	− 1.362	£64,269^a^

SOT-RRMS	Alemtuzumab			Fingolimod			Daclizumab		
	Inc. cost	Inc. QALYs	ICER	Inc. cost	Inc. QALYs	ICER	Inc. cost	Inc. QALYs	ICER
Company base case	− £17,549	0.153	Dominant	− £72,065	0.944	Dominant	− £66,397	0.548	Dominant
ERG scenario (R1, R2–R6)	− £8711	0.004	Dominant	− 6642	0.013	Dominant	− £7749	0.010	Dominant

*R1* for 6-month CDP, cladribine is no more effective than placebo

For qualifying ARR, the effectiveness of alemtuzumab and daclizumab are set equal to the effectiveness of cladribine where appropriate; R2 = waning effect: the effectiveness of cladribine is set to 75% between years 2 and 4; R3 = no re-exposure to cladribine or alemtuzumab; R4 = treatment discontinuation only at EDSS state 7 after 2 years; R5 = TA32 EDSS state costs [34]; R6 = no carer disutility

*ARR* annualised relapse rate, *CDP* confirmed disability progression, *ICER* incremental cost-effectiveness ratio, *Inc.* incremental, *QALY* quality-adjusted life-year

^a^The ICER represents the monetary gain per QALY lost rather than the cost per QALY gained

### Conclusions of the Evidence Review Group Report

The company presented direct clinical effectiveness evidence (cladribine vs. placebo) from the CLARITY trial. This trial was of good quality and was well conducted. The RES-RRMS and SOT-RRMS subgroups and three outcomes (NEDA-3, time to 6-month CDP and proportion of patients with 6-month CDP) were defined retrospectively. The ERG considers that the post-hoc definitions and analyses were necessary to address the final scope issued by NICE, but the sample size for the SOT-RRMS subgroup was too small to detect a statistically significant difference for all outcomes. For the indirect evidence, the ERG noted that the results of the NMAs carried out by the company should be viewed with caution owing to the small number of studies that were available for the key efficacy outcomes. The ERG also remained unconvinced that the results of the meta-regression presented by the company were valid.

The effect of DMTs on slowing disability progression (6-month CDP) was the main cost-effectiveness driver in the company’s economic model. On this, for the RES-RRMS subgroup, the ERG and the company agreed that treatment cladribine was statistically significantly more effect than placebo and no more effective than treatment with other DMTs. However, the ERG did not agree with the company’s decision to apply a lower waning effect and discontinuation rates to cladribine compared to those applied to treatment with other DMTs. The differential rates applied were not based on evidence and overestimated the cost effectiveness of cladribine. There was no statistically significant evidence of effectiveness of cladribine over placebo in the SOT-RRMS subgroup for either 6-month CDP or qualifying ARR. There was therefore no basis on which to undertake an economic analysis. Overall, given the substantial uncertainties about the long-term prognosis, in terms of disability progression and ARR, for patients treated with DMTs, the ERG considered that any economic results produced by the company model, even after ERG modifications, should be treated with caution.

## National Institute for Health and Care Excellence

The AC reviewed the evidence available on the clinical and cost effectiveness of cladribine alongside testimony from clinical experts and patient representatives.

### Clinical Need and Patient Perspective

The AC heard from a patient expert that relapses and residual disability between relapses can substantially limit a patient’s ability to work. The AC concluded that frequent hospital appointments for drug treatment and monitoring cause significant disruption to patients’ lives and careers. The AC also recognised the benefit of an oral treatment taken in two short courses over 2 years.

### Current Practice and Comparators

The AC heard from clinical experts that many patients with MS do no take DMTs, but patients with highly active disease receive DMTs. The AC also heard from the clinical experts that clinicians follow NICE guidance that recommends that patients with RES-RRMS receive alemtuzumab or natalizumab. Similarly, patients with SOT-RRMS receive alemtuzumab or fingolimod. At the time of the AC meeting, NICE guidance had recommended daclizumab for patients with RES-RRMS and SOT-RRMS if alemtuzumab is contraindicated or otherwise unsuitable. Clinical experts explained that patients with RES-RRMS are clinically identifiable, but patients with SOT-RRMS are more difficult to define. The AC heard from the experts that the company had defined the SOT-RRMS subgroup based on the number of MRI lesions. The clinical experts noted that it was not the number of, but the increase in, MRI lesions that is important for measuring response to treatment. The AC, therefore, agreed to refer to MRI evidence of disease activity rather than use the company’s definition of suboptimal treatment. The AC concluded that the RES-RRMS and SOT-RRMS subgroups were representative of the population who would receive cladribine. The AC was satisfied that the company had correctly specified relevant comparators for the RES-RRMS and SOT-RRMS subgroups.

### Clinical Effectiveness

The AC agreed with the ERG that evidence from the CLARITY trial showed that, compared with placebo, treatment with cladribine statistically significantly reduced the number of relapses experienced, and marginally, although not statistically significantly, delayed progression for patients in the RES-RRMS subgroup. The AC also considered that treatment with cladribine was statistically significantly more effective than placebo in terms of ARR, 3-month CDP and 6-month CDP in the HDA-RRMS subgroup, a group that includes the RES-RRMS and SOT-RRMS subgroups. The AC, therefore, believed that the result in the HDA-RRMS is generalisable to the RES-RRMS and SOT-RRMS subgroups.

The AC noted that, where comparative results were available from indirect analyses, the confidence intervals from the NMAs were wide and overlapped between all treatments for patients in the RES-RRMS and SOT-RRMS subgroups. The AC heard from the ERG that the credible intervals in the NMA and meta-regression overlapped for all treatments for patients in the RES-RRMS and SOT-RRMS subgroups. The AC then concluded that the effectiveness of cladribine was not statistically significantly different from the effectiveness of alemtuzumab, daclizumab, fingolimod or natalizumab in the RES-RRMS and SOT-RRMS subgroups based on results from the NMA and meta-regression. It also noted that the estimated effectiveness of cladribine, compared with placebo, generated by the meta-regression for the RES-RRMS and SOT-RRMS subgroups was similar to the estimates from the NMA. The AC was concerned that the meta-regression analysis underestimated the efficacy of alemtuzumab compared with placebo. The company suggested this could be explained by the differences in baseline risk between trials. The company then compared the effect sizes predicted by the meta-regression with the effect sizes seen in the studies that assessed alemtuzumab in the NMAs. The AC agreed with the ERG’s concerns that there were differences in effect sizes that are not explained by differences in baseline risk, which would make the company’s approach invalid. The AC acknowledged the company’s attempts to address the data limitations but, on balance, agreed with the ERG that the meta-regression approach may be invalid.

### Cost Effectiveness

The AC agreed that the company’s model was appropriately structured and that most of the assumptions were generally reasonable. The AC accepted most of the ERG’s amendments to the company’s base-case model but noted that the changes did not make a substantial difference to the size of the company’s base-case cost effectiveness estimates. The ERG had argued for the exclusion of the utility decrement for carers, but the AC was willing to consider the impact of MS on carers and on carers’ HRQoL in the cost-effectiveness analysis. The ERG updated its amendments to reflect the AC’s preference. The AC noted that, in isolation, none of the ERG’s amendments to the company model changed the company’s base-case results, as, for both subgroups, treatment with cladribine continued to dominate all other treatments. As a result of implementing all of the ERG’s amendments to the company model, treatment with cladribine remained more effective and cheaper than daclizumab, fingolimod or natalizumab for the relevant subgroups. Treatment with cladribine was less effective and cheaper than alemtuzumab for both the RES-RRMS and SOT-RRMS subgroups, resulting in ICERs of £219,549 gained per QALY lost and £372,802 gained per QALY lost respectively.

### Final Guidance

The AC recommended cladribine as a treatment option for patients with RES-RRMS and SOT-RRMS. The final guidance was published by NICE in December 2017 [3].

## Conclusion

The main issues ecountered during this STA stermed from the post-hoc definition of subsubgroups and clinical outcomes. The evidence provided by the company focused on the use of cladribine for patients with RES-RRMS and SOT-RRMS. The company had expected a marketing authorisation from the EMA for the use of cladribine in patients with HDA-RRMS, for which RES-RRMS and SOT-RRMS are subgroups, yet the company did not provide any economic evidence for patients with HDA-RRMS.

The effect of DMTs on slowing disability progression was the largest driver of cost effectiveness in the economic model. Sample sizes for the RES-RRMS and SOT-RRMS subgroups were small, thus there was insufficient direct clinical effectiveness evidence for the ERG to conclude that cladribine was better than placebo in reducing disease progression. The indirect evidence was also weak for the subgroups. However, both the direct and indirect evidence showed that treatment with cladribine was statistically significantly better than placebo in delaying disease progression and in reducing the relapse rate. The AC made the decision to recommend the use of cladribine for patients with RES-RRMS and SOT-RRMS based on the clinical effectiveness result in the HDA-RRMS population, for which an economic evidence should have been provided.
